# Fast Detection of Uric Acid in Urine for Early Diagnosis Using THz Polarized Waves

**DOI:** 10.3390/s25041004

**Published:** 2025-02-08

**Authors:** Zahra Mazaheri, Giorgia Federico, Can Koral, Gian Paolo Papari, Lakshmi Ullatil, Paolo Russo, Antonello Andreone

**Affiliations:** 1 Department of Physics “Ettore Pancini”, University of Naples Federico II, I-80126 Naples, Italy; gianpaolo.papari@unina.it (G.P.P.); lakshmiullatil00@gmail.com (L.U.); paolo.russo@unina.it (P.R.); 2 Naples Unit, National Institute for Nuclear Physics (INFN), I-80126 Naples, Italy; can.koral@unibas.it; 3 Department of Molecular Medicine and Medical Biotechnology, University of Naples Federico II, I-80131 Naples, Italy; giorgia.federico@unina.it; 4 Department of Health Sciences, University of Basilicata, I-85100 Potenza, Italy

**Keywords:** urinary biomarker, uric acid, THz spectroscopic ellipsometry

## Abstract

Towards new and improved techniques in liquid biopsy for the diagnosis of diseases, this study reports experimental evidence of a rapid and reliable method based on terahertz (THz) time-domain spectroscopic ellipsometry (TDSE) for the early diagnosis of kidney-related diseases, using the detection of uric acid (UA) content in urine. Employing a custom-built THz-TDSE system, we analyzed the absorption and dispersion response of synthetic urine samples with varying concentrations of UA. The technique provides a prompt indication of UA presence and concentration, thanks to the sensitivity of THz waves to intermolecular interactions such as hydrogen bonding. The results clearly show a linear correlation between the UA concentration and changes in the absorption spectra of urine in the frequency window 0.2–1.2 THz, with the minimum detectable UA concentration being approximately close to the upper limit of normal UA levels in urine. The increase in the absorption coefficient as a function of the UA concentration provides a means for a quantifiable measure of the UA biomarker in urine for assessing disease stage. This study proves that THz-TDSE is capable of detecting UA at concentrations relevant for early-stage diagnosis of renal diseases, with an estimated sensitivity of 0.2 g/L in the region where the material response is linear.

## 1. Introduction

Uric acid (UA) is a metabolic waste product in the body. When a chemical called purine breaks down, UA is produced and dissolved in the blood, where its non-pathological serum levels are below 6 mg/dL (in women) and 7 mg/dL (in men). After passing through the kidneys (two-thirds of the total amount, one-third being excreted by the gut), it is eliminated from the body by urination. A thorough database has been recently compiled by the U.S. Department of Agriculture (USDA), reporting the presence of purine in various foods and drinks [1]. The ideal amount of UA in urine is 0.1–0.5 g/L, considering that an individual produces 1–2 L of urine per day [2] and that typically over 600 mg UA/day are excreted [3]. Values both above (hyperuricosuria) and below normal levels in urine have been linked to a number of inflammatory states, providing evidence that UA is actually considered as a biomarker for several diseases. Indeed, abnormally high uric acid levels have been correlated with gout [4], hypertension [5], and cardiovascular [6] and renal diseases [7]. Early detection of renal diseases, such as bladder cancer, is crucial for improving treatment outcomes and survival rates. Traditional cancer screening methods include imaging techniques [8], biopsies [9], and blood tests [10]. In recent years, liquid biopsy has emerged as a minimally invasive detection method for cancer molecular profiling not only of blood but also of other body fluids, including urine. Indeed, recent advances in medical research have highlighted the potential of biomarkers, such as UA levels in urine, to serve as early indicators of cancer [11]. Traditional methods for UA detection in urine often rely on chemical analysis including enzymatic assays, colorimetric assays including fluorescence methods [12], and chromatography, but they also include electrochemical detection, surface plasmon resonance detection, and capillary electrophoresis [13]. These methods are usually accurate, however, they require extensive sample preparation, well-controlled laboratory settings, and high-quality reagents, making them time-consuming and expensive [14]. On the other hand, optical methods like UV-Vis spectroscopy, fluorescence, surface plasmon resonance, and Raman spectroscopy, while offering high sensitivity for detecting UA at low concentrations, are often very sensitive to environmental conditions, such as temperature, pH, and light scattering, which can affect their accuracy and reliability [15,16]. Terahertz (THz) spectroscopy, which operates in the frequency range of 0.1–10 THz, has emerged as a promising and robust tool for biomolecular analysis [17] due to its sensitivity to molecular vibrations and low-energy transitions [18]. THz spectroscopy represents a specific recent investigative goal in the research on metabolic biomarkers in urine liquid samples, for early cancer detection via so-called liquid biopsy. In this context, specific spectral fingerprints in the THz regime of biomolecules dissolved in urine have been investigated via various THz technologies for oncological disease detection [2]. Biosensors (such as metasurfaces [19,20]) and microfluidic techniques are also emerging in the field, showing great potential for fast real-time analysis in liquid biopsy, which use electrochemical properties to detect biomarkers in urine samples [21,22]. These techniques, apart from adding additional complexity to measurement, can be combined with THz-TDS techniques to improve the detection sensitivity.

Table 1 summarizes the existing techniques in liquid biopsies and highlights the principles, advantages, and drawbacks for each of them.

Although direct studies on UA detection in urine using THz spectroscopy are limited, the potential for this application has been suggested in the diagnosis of various diseases [2]. THz radiation is sensitive to molecular vibrations and intermolecular interactions [23], which is a key factor in studying biomarkers such as UA in urine. Using conventional spectroscopic techniques in the THz region such as time-domain transmission spectroscopy can be challenging for this purpose, due to the strong signal absorption of urine. For this aim, researchers have used techniques such as freeze-drying or concentrating the samples to enhance the detection of UA, which however may require excessive sample amounts [24]. Conversely, novel techniques such as THz ellipsometry, being a self-reference and surface-related technique, overcomes the above-mentioned challenge and removes the barrier towards a fast and accurate method for detecting urinary biomarkers such as UA with high precision. The THz spectroscopy technique has been already used to detect protein concentrations in urine, linked to kidney diseases [25], glucose concentrations linked to diabetes [26], and bacteria detection as a sign of urinary infection [27]. All these research studies show the potential of THz spectroscopic techniques in liquid biopsy.

In this work, we demonstrate the potential of an experimental tool based on THz time-domain spectroscopic ellipsometry (TDSE) for fast and quantitative liquid biopsy diagnosis of kidney-related diseases, using UA detection in urine, as a first attempt to move from research focused methods towards practical clinical applications.

## 2. Materials and Methods

### 2.1. Sample Preparation

Uric acid powders (BioXtra) with a purity of 99.7% and synthetic urine (Sigmatrix) with a pH ranging between 6.5 and 7.2 were purchased from Merck Life Science, Darmstadt, Germany. Synthetic urine is formulated to closely replicate human urine, which is a water solution comprising calcium chloride, magnesium chloride, potassium chloride, sodium chloride, sodium phosphate, sodium sulfate, urea, and creatinine. Before testing urine solutions with different UA concentrations, we first prepared 1 mm thick pellets by mixing uric acid and high-density polyethylene (Merck Life Science, Darmstadt, Germany) with a ratio of 1:1 (*w*/*w*) and pressing the dried powders at 10 tons for 2 min. For the liquid samples fabrication, we started from a stock solution with a uric acid concentration of 50 g/L by using an analytical balance (Sartorius, Göttingen, Germany) with a resolution of 0.0001 g. The stock solution was then diluted to create concentrations of 1 g/L, 2 g/L, and 3 g/L of uric acid in urine. All samples were prepared in controlled and sterile conditions to avoid contamination. For each test, 5 mL of each solution was placed in a Petri dish with 14 mm depth and 50 mm diameter. The container was deep enough to make the unavoidable secondary time signals produced by the THz beam reflected from the lower liquid–dish interface negligible. Following preparation, the samples were stored at 4 °C. Before measurements, they were kept under ambient conditions for approximately two hours to reach room temperature.

### 2.2. Experimental Setup

**Figure 1:**
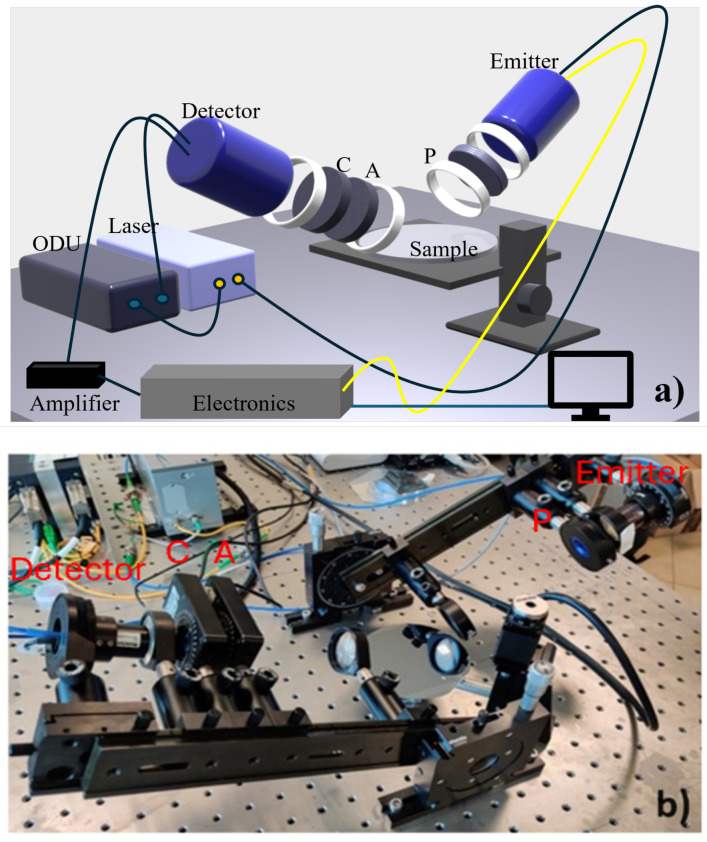
(**a**) 3D rendering and (**b**) picture of the home-built THz-TDSE setup. In (**a**) C, A, and P represent the conductive wire-grid polarizers, where C = compensator, A = analyzer, and P = polarizer.

We utilized a custom-built THz time-domain ellipsometer (Figure 1), featuring a femtosecond laser source fiber-coupled to two photoconductive antennas for coherent THz pulse emission and detection. Polymeric TPX (polymethylpentene) lenses are used to collimate and focus the THz pulse. Three conductive wire grid polarizers (WGPs), marked by P, A, and C in Figure 1, are employed to control, detect, and compensate the polarization state, respectively. This setup, compared to previous designs, provides enhanced precision and reliability [28]. Its flexible design and accurate calibration, mentioned in detail in our previous work [24], enable precise probing of liquid polarization responses in the sub THz range. The opto-mechanical design permits changing the incident angle in a wide range ($10^{\circ }<\theta \leq 90^{\circ }$) with an accuracy of $0.5^{\circ }$. The THz spot size at the focal length of focusing lenses is approximately 5 mm. Further details on the measurement setup can be found in [29]. In time-domain ellipsometric measurements, the p-polarized and s-polarized electric fields reflected from the sample surface are recorded [30]. Afterward, we obtained frequency-dependent information on both the amplitude and phase of the reflected signals using fast Fourier transform (FFT) analysis, from which we directly retrieved the ellipsometric parameters (Ψ;Δ) defined as the amplitude ratio and the phase difference of the p- and s-polarized states, respectively. The ellipsometric parameters (Ψ;Δ) are linked to the complex dielectric response of the material under test [31]:

(1) $$\varepsilon^{\prime }=\sin^{2}\theta \left[1+\frac{\tan^{2}\theta \left(\cos^{2}2\Psi -\sin^{2}2\Psi \sin^{2}\Delta \right)}{{\left(1+\sin2\Psi \cos\Delta \right)}^{2}}\right]$$

(2) $$\varepsilon^{\prime \prime }=\sin^{2}\theta \frac{\tan^{2}\theta \sin4\Psi \sin\Delta }{{\left(1+\sin2\Psi \cos\Delta \right)}^{2}}.$$

where θ is the incident angle of the beam, *n* and *k* represent the refractive index and the extinction coefficient, respectively.

Ellipsometric data have the highest precision in evaluating the optical response in the vicinity of the Brewster angle (for almost transparent materials) or pseudo-Brewster angle (for lossy materials) $\theta_{B}$ [31]. In our setup it can be shown that in measuring the ellipsometric angles (Ψ and Δ) a minimum deviation occur (0.5° and 0.05° respectively) close to $\theta_{B}$ [18]. This translates into a maximum relative error in the retrieved values of the real and imaginary parts of material optical properties (i.e., the refractive index *n* and the absorption coefficient *k*) of around 2% and 5%, respectively. The error evaluation has been described in details in [18]. Consequently, θ is set at $65^{\circ }$, close to the pseudo-Brewster angle $\theta_{B}$ of pure synthetic urine (≃68° in this frequency range). Using TDSE we can therefore achieve an accuracy comparable to chemical methods [2]. It is worth mentioning that the error in concentration estimation is by far smaller than the error inherent in the technique itself. All measurements were conducted under ambient conditions at a controlled temperature (approximately 25 °C.

The entire process, from the signal emission and detection to the end of the retrieval procedure to get the dielectric response of the liquid sample, takes less than a minute. At good reason therefore TDSE can be considered a fast technique for the uric acid detection in urine.

## 3. Results and Discussion

We first measured the optical response of uric acid in powder form by examining the mixed UA/PE (1:1 *w*/*w*) pellets. To this aim, we used the time-domain spectroscopic setup in transmission mode, with the THz beam normally impinging on the sample surface. To retrieve from the mixture the complex dielectric function of pure UA, ${\tilde{\varepsilon }}_{UA}$, we applied a Mean Field Theory (MFT) approach using the Landau formula [32].

(3) $${\tilde{\varepsilon }}_{UA}=\frac{{\left[{\tilde{\varepsilon }}_{eff}^{-3}-{\tilde{\varepsilon }}_{PE}^{-3}\right]}^{3}}{\sigma_{UA}^{3}}$$

Here, ${\tilde{\varepsilon }}_{eff}$ and ${\tilde{\varepsilon }}_{PE}$ are the complex dielectric function of the mixed medium (pellet) and polyethylene, respectively, whereas the parameter $\sigma_{UA}={\left[1+\frac{\rho_{UA}\left(1-a_{UA}\right)}{\rho_{PE}a_{UA}}\right]}^{-1}$ is related to the density of UA ($\rho_{UA}$), density of PE ($\rho_{PE}$), and weight fraction of UA ($a_{UA}$) in the mixed medium.

**Figure 2:**
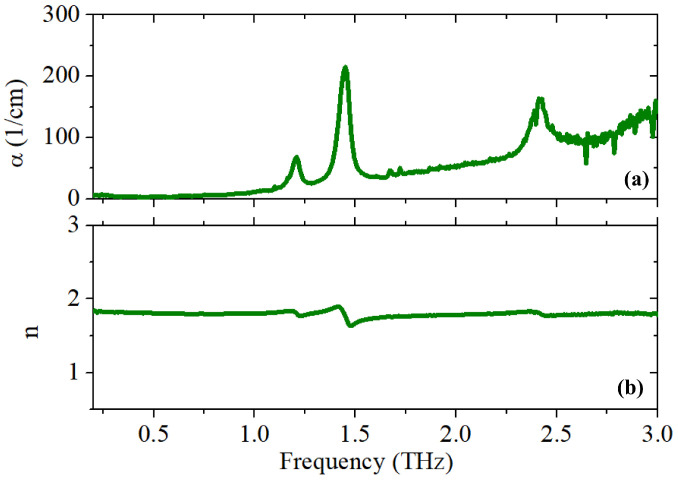
(**a**) The absorption coefficient α and (**b**) refractive index *n* vs. frequency for pure UA extracted by applying the MFT approach to the mixed pellet of UA/PE (1:1 *w*/*w*).

The refractive index *n* and absorption α (directly linked to the absorption coefficient *k* through the relation α=4πk/λ, λ being the electric field wavelength) frequency spectra of pure UA are shown in Figure 2a and Figure 2b, respectively. As illustrated in the graphs, uric acid has two strong and close absorption peaks at around 1.2 and 1.5 THz, which are related to the out-of-plane and torsional vibrational modes, respectively, in the uric acid cluster [33]. A third distinct peak is observed at around 2.4 THz, possibly originating from a further intermolecular vibrational mode at a higher frequency [34].

**Figure 3:**
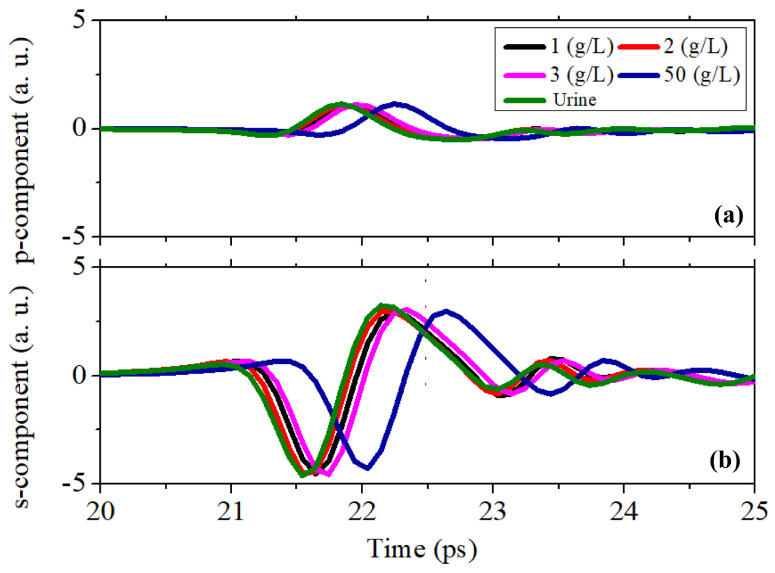
The time-domain signals for (**a**) the p-polarized component and (**b**) the s-polarized component reflected from the urine solution with different concentrations of uric acid.

After this preliminary investigation, a set of liquid urine samples with uric acid was tested using the setup in an ellissometric configuration. Figure 3a,b show, respectively, the time-dependent p- and s-polarized components of the electric field reflected from the surface of different liquid samples. The increase in the refractive index produces a shift to the right in the time scale for both components, caused by the increased optical path. The increase in the UA concentration in urine instead reduces the signal intensity of the s-component only, whereas the p-polarized component is almost constant for all samples. This reveals the fact that adding UA to urine does not substantially modify its conductivity [31].

**Figure 4:**
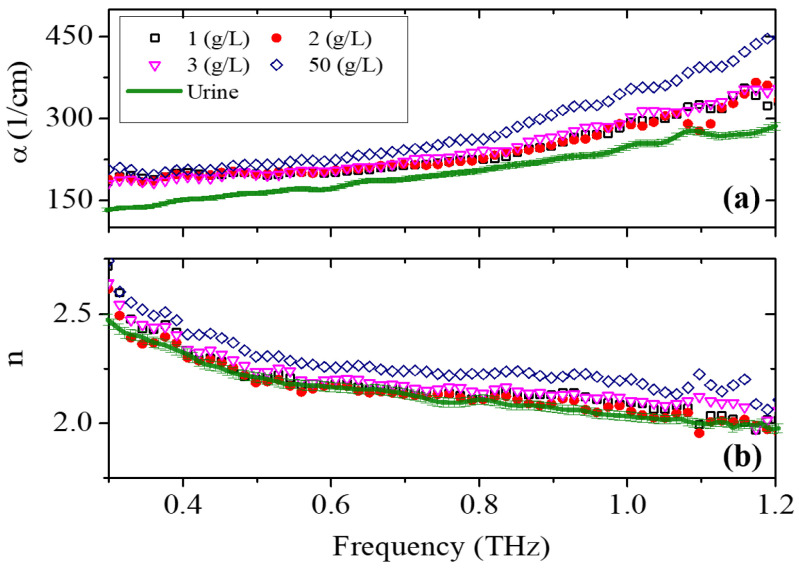
(**a**) The absorption and (**b**) the dispersion frequency spectrum of urine solutions with concentrations of 0, 1, 2, 3, 50 g/L of uric acid.

Again, absorption and dispersion were measured as a function of frequency in the 0.3–1.2 THz spectral window with 0.007 THz resolution at different concentrations of UA in urine (Figure 4).

The frequency dependence of the pure urine sample clearly resembles the behavior observed in pure water [35]. The reduction in the frequency bandwidth with respect to the pellet sample is due to the presence of water, which is highly absorptive in the THz region, making the signal-to-noise ratio (SNR) extremely low. Adding UA to the solution (1, 2, 3 or 50 g/L) results in an increase in both α and (to a lesser extent) *n*. This is given by the specific ability of uric acid to form hydrogen bonds with different components of urine, such as urea and salts dissolved in solution [36,37,38].

Consistent with common practice in the literature, our analysis will focus on the absorption spectra of different solutions to maintain standard terminology and facilitate a comparison with existing studies. Using this technique, the minimum detectable concentration of UA in urine is approximately close to the upper limit of the normal UA concentration in 1 L of urine. Consequently, this method shows promise for the early-stage diagnosis of renal diseases.

**Figure 5:**
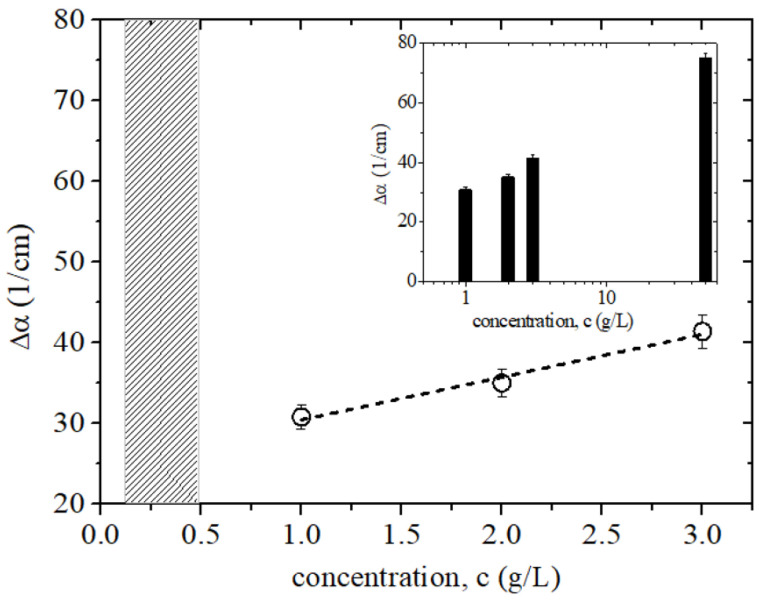
The variation Δα in the urine solution as a function of the UA concentration for levels slightly exceeding the physiological concentration. Presented data are the mean value of four frequency points (0.6, 0.7, 0.9, and 1 THz). The hatched area represents instead the range of physiological levels. The dashed line shows the linear dependence of the change in absorption as a function of concentration. In the inset, the same quantity is plotted on a semi-log scale to show its change over the full range of the UA concentration.

Deviation from the urine absorption by adding UA ($\Delta \alpha =\alpha_{solution}-\alpha_{urine}$) is shown in Figure 5, indicating a clear and significant relationship between the concentration and absorption coefficient; this emphasizes that as the concentration of uric acid increases, Δα also increases. Immediately above the limit of the UA physiological level (0.6–0.7 g/L), there is already a consistent change in the absorption values in the overall frequency range investigated. The observed behavior can be employed to quantify the severity of renal diseases. Presented data comprise the mean value of four frequency points (0.6, 0.7, 0.9, and 1 THz). In the inset, the same data are reported in a histogram plot and on a semi-log scale, together with the highest concentration level given by the stock solution.

The dependence of the UA concentration on the change in the absorption response is clearly linear in the low concentration region. Therefore, we can estimate the UA concentration in urine based on its deviation Δα from the absorption properties of pure urine, representative of a healthy clinical baseline. This approach allows for a quantifiable estimation of the stage of renal disease. The slope of the linear fit can be considered as a quantity, which links the experimentally observed Δα to estimate the UA concentration (c) and hence the disease stage, as follows:

(4) S=Δα/ΔC

Data displayed in Figure 5 show that by using such a simple, reference-free THz technique, we can quickly measure the content of UA in urine, starting from approximate physiological levels to a high level of UA in urine and specifically targeting the detection of early-stage renal disease (for concentration higher than 1 g/L). Since the minimum measurable change in absorption is estimated to be in the order of cm^−1^ [28], data show that in the linear range, the technique reaches a sensitivity in the detection of uric acid of approximately 0.2 g/L. The inset presents data for all concentrations, including the stock solution, showing that the technique is a reliable tool for detecting hyperuricosuria, which can be indirectly correlated with different inflammatory states.

The method offers a reasonable sensitivity with less than a 5% error in the evaluation of solutions’ absorption responses, which is comparable with the sensitivity of traditional techniques while providing several non-trivial advantages, such as the following: simpler, minimally time-consuming, cost-effective and less prone to environmental conditions [31].

## 4. Conclusions

We introduce the THz ellipsometry technique as a fast, self-referenced, and accurate alternative to traditional chemical and optical techniques for detecting uric acid as a urinary biomarker. Time-domain spectroscopy is a label-free and cost-effective technique; it does not require sophisticated instrumentation, specialized personnel or specific sample preparation; and it can be easily employed for routine clinical measurements. Our findings reveal that due to the sensitivity of the technique to molecular vibrations, H-bond formation signatures, and low-energy transitions, THz-TDSE offers a unique combination of non-destructive analysis, sensitivity to molecular dynamics, high-resolution spectroscopy, and comprehensive optical characterization, making it a powerful tool for early-stage disease diagnosis. Its advantages over traditional chemical and optical techniques include a fast process, deeper penetration, and detailed structural information. These features make THz-TDSE particularly well suited for biomedical applications where detailed, non-destructive analysis of biological samples is crucial, as in case of urinary biomarkers detection [39]. The experimental results show a clear correlation between the UA concentration and changes in the absorption spectra, which can be estimated by the slope of a linear fit of the concentration–response curve, validating the potential of this method for the early-stage recognition and stage determination of renal diseases. The analysis of the refractive index and absorption spectra indicates that as the concentration of UA increases, so does the absorption coefficient. This trend sheds light on the capability of THz ellipsometry in quantifying the UA concentration in urine. The method needs minimal sample preparation and is easy to carry out in short time with high reliability. All these factors promote its practical adoption in clinical applications. In addition, the technique achieves a detection sensitivity close to the upper limit of normal UA concentrations in urine. This makes it a promising tool for the non-invasive and rapid monitoring of early-stage kidney conditions like gout, hyperuricemia, and kidney stones. In a broader context, our THz ellipsometry-based approach for uric acid detection complements similar emerging non-invasive diagnostic methods, such as microwave reflectometry imaging for in vivo skin cancer detection [38]. There is a growing body of evidence that novel electromagnetic wave-based technologies have the potential to enhance the speed, accuracy, and accessibility of medical diagnostics, especially in the mm wave region where many free-space measurement systems already exist [40]. The possibility to use various frequency ranges can provide complementary insights into material properties and underscores the potential for cross-disciplinary advancements in diagnostic and material characterization technologies.

## Figures and Tables

**Table 1:** Comparison of chemical, optical, and THz spectroscopic techniques for uric acid detection.

Method	Principle	Advantages	Disadvantages
**Chemical**	Based on chemical reactions, such as enzymatic or colorimetric assays	High sensitivity Reliable if done under well-established protocols	Complicated sample preparation Time-consuming Expensive
**Optical **	UV-Vis absorption, fluorescence, or Raman spectroscopy	Sensitive to low concentrations Provides molecular insights	Sensitive to environmental factors Affected by light scattering
**THz-TDS **	Investigate the complex refractive index (dispersion and absorption) in the THz range	Non-destructive Minimal preparation Cost-effective Rapid analysis	Lower sensitivity compared to chemical techniques Need for validation in real-world cases

## Data Availability

The raw data supporting the conclusions of this article will be made available by the authors upon request.

## References

[1] Heydorn K.C., Pehrsson P.R., Wu X., Dwyer J.T., Ershow A.G., Wambogo E., Gahche J., Hord N., Hays F., Garrett T.J. (2024). USDA/NIH-ODS Special Interest Databases for Dietary Supplements and Foods: Bridging Data Gaps for Dietary Health. Curr. Dev. Nutr..

[2] Abina A., Korošec T., Puc U., Jazbinšek M., Zidanšek A. (2023). Urinary metabolic biomarker profiling for cancer diagnosis by terahertz spectroscopy: Review and perspective. Photonics.

[3] Adomako E., Moe O.W. (2020). Uric acid and urate in urolithiasis: The innocent bystander, instigator, and perpetrator. Semin. Nephrol..

[4] Feig D.I., Kang D.H., Johnson R.J. (2008). Uric acid and cardiovascular risk. N. Engl. J. Med..

[5] Lanaspa M.A., Andres-Hernando A., Kuwabara M. (2020). Uric acid and hypertension. Hypertens. Res..

[6] Madero M., Sarnak M.J., Wang X., Greene T., Beck G.J., Kusek J.W., Collins A.J., Levey A.S., Menon V. (2009). Uric acid and long-term outcomes in CKD. Am. J. Kidney Dis..

[7] Hegerty K., Jaure A., Scholes-Robertson N., Howard K., Ju A., Evangelidis N., Wolley M., Baumgart A., Johnson D.W., Hawley C.M. (2023). Australian Workshops on patients’ perspectives on hemodialysis and incremental start. Kidney Int. Rep..

[8] Lam S., MacAulay C., Palcic B. (1993). Detection and localization of early lung cancer by imaging techniques. Chest.

[9] Allison J.E., Sakoda L.C., Levin T.R., Tucker J.P., Tekawa I.S., Cuff T., Pauly M.P., Shlager L., Palitz A.M., Zhao W.K. (2007). Screening for colorectal neoplasms with new fecal occult blood tests: Update on performance characteristics. J. Natl. Cancer Inst..

[10] Cohen J.D., Li L., Wang Y., Thoburn C., Afsari B., Danilova L., Douville C., Javed A.A., Wong F., Mattox A. (2018). Detection and localization of surgically resectable cancers with a multi-analyte blood test. Science.

[11] Smith E.M., Beresford M.W. (2017). Urinary biomarkers in childhood lupus nephritis. Clin. Immunol..

[12] Lakowicz J. (2006). Principles of Fluorescence Spectroscopy.

[13] Wang Q., Wen X., Kong J. (2020). Recent progress on uric acid detection: A review. Crit. Rev. Anal. Chem..

[14] Tian Y., Fan X., Chen K., Chen X., Peng W., Wang L., Wang F. (2024). Optical biomarker analysis for renal cell carcinoma obtained from preoperative and postoperative patients using ATR-FTIR spectroscopy. Spectrochim. Acta Part Mol. Biomol. Spectrosc..

[15] Markelz A., Roitberg A., Heilweil E.J. (2000). Pulsed terahertz spectroscopy of DNA, bovine serum albumin and collagen between 0.1 and 2.0 THz. Chem. Phys. Lett..

[16] Zhou J.W., Zheng X.B., Liu H.S., Wen B.Y., Kou Y.C., Zhang L., Song J.J., Zhang Y.J., Li J.F. (2024). Reliable quantitative detection of uric acid in urine by surface-enhanced Raman spectroscopy with endogenous internal standard. Biosens. Bioelectron..

[17] Koral C., Mazaheri Z., Papari G.P., Andreone A., Drebot I., Giove D., Masullo M.R., Mettivier G., Opromolla M., Paparo D. (2022). Multi-pass free electron laser assisted spectral and imaging applications in the terahertz/far-IR range using the future superconducting electron source BriXSinO. Front. Phys..

[18] Mazaheri Z., Koral C., Andreone A., Marino A. (2022). Terahertz time-domain ellipsometry: Tutorial. JOSA A.

[19] Papari G.P., Koral C., Andreone A. (2019). Encoded-enhancement of THZ metasurface figure of merit for label-free sensing. Sensors.

[20] Wu M., He X., Lu G., Geng Z., Zhang Y. (2024). Multi-mode non-diffraction vortex beams enabled by polarization-frequency multiplexing transmissive terahertz metasurfaces. J. Appl. Phys..

[21] Sequeira-Antunes B., Ferreira H.A. (2023). Urinary biomarkers and point-of-care urinalysis devices for early diagnosis and management of disease: A review. Biomedicines.

[22] Hwang C., Lee W.J., Kim S.D., Park S., Kim J.H. (2022). Recent advances in biosensor technologies for point-of-care urinalysis. Biosensors.

[23] Mazaheri Z., Papari G.P., Andreone A. (2024). Dielectric Response of Different Alcohols in Water-Rich Binary Mixtures from THz Ellipsometry. Int. J. Mol. Sci..

[24] Mazaheri Z., Koral C., Andreone A. (2022). Accurate THz ellipsometry using calibration in time domain. Sci. Rep..

[25] Xue Z., Mao P., Peng P., Yan S., Zang Z., Yao C. (2023). Terahertz spectra of proteinuria and non-proteinuria. Front. Bioeng. Biotechnol..

[26] Emaminejad H., Mir A., Farmani A. (2021). Design and simulation of a novel tunable terahertz biosensor based on metamaterials for simultaneous monitoring of blood and urine components. Plasmonics.

[27] Yu W., Shi J., Huang G., Zhou J., Zhan X., Guo Z., Tian H., Xie F., Yang X., Fu W. (2022). THz-ATR spectroscopy integrated with species recognition based on multi-classifier voting for automated clinical microbial identification. Biosensors.

[28] Huang Q., Liu W., Han M., Yang Z., Liu J., Wang K. (2024). Terahertz ellipsometry based on the long-distance diffraction-free beam. Opt. Lasers Eng..

[29] Ngai K. (2018). Interpretation of the GHz to THz dielectric relaxation dynamics of water in the framework of the coupling model. J. Mol. Liq..

[30] Cai Z., Zhu C., Chen G., Wu Y., Gu J., Ma C., Gao H., Li L., Guo S. (2023). Study on intermolecular hydrogen bond of uric acid water-clusters. Chem. Phys. Lett..

[31] Fujiwara H. (2007). Spectroscopic Ellipsometry: Principles and Applications.

[32] Banhegyi G. (1988). Numerical analysis of complex dielectric mixture formulae. Colloid Polym. Sci..

[33] Chen T., Zhang Q., Li Z., Hu F. (2020). Intermolecular weak interactions of crystalline purine and uric acid investigated by terahertz spectroscopy and theoretical calculation. J. Lumin..

[34] Upadhya P., Shen Y., Davies A., Linfield E. (2003). Terahertz time-domain spectroscopy of glucose and uric acid. J. Biol. Phys..

[35] Wu X., Tao R., Zhang T., Liu X., Wang J., Zhang Z., Zhao X., Yang P. (2023). Biomedical applications of terahertz spectra in clinical and molecular pathology of human glioma. Spectrochim. Acta Part Mol. Biomol. Spectrosc..

[36] Cataldo A., Cino L., Distante C., Maietta G., Masciullo A., Mazzeo P.L., Schiavoni R. (2024). Integrating microwave reflectometry and deep learning imaging for in-vivo skin cancer diagnostics. Measurement.

[37] Liu X., Gan L., Yang B. (2021). Millimeter-wave free-space dielectric characterization. Measurement.

[38] Burtis C.A., Ashwood E.R. (1999). Tietz textbook of clinical chemistry. Philadelphia.

[39] Coe F.L., Coe F. (1983). Uric acid and calcium oxalate nephrolithiasis. Kidney Int..

[40] Armenta-Castro A., Núñez-Soto M.T., Rodriguez-Aguillón K.O., Aguayo-Acosta A., Oyervides-Muñoz M.A., Snyder S.A., Barceló D., Saththasivam J., Lawler J., Sosa-Hernández J.E. (2024). Urine biomarkers for Alzheimer’s disease: A new opportunity for wastewater-based epidemiology?. Environ. Int..

